# Assessment of transition readiness in adolescents in Thailand with rheumatic diseases: a cross-sectional study

**DOI:** 10.1186/s12969-021-00602-5

**Published:** 2021-06-30

**Authors:** Sirinthip Kittivisuit, Butsabong Lerkvaleekul, Sirisucha Soponkanaporn, Pintip Ngamjanyaporn, Soamarat Vilaiyuk

**Affiliations:** 1 Division of Rheumatology, Department of Pediatrics, Faculty of Medicine Ramathibodi Hospital, Mahidol University, 270 Rama VI Road, Ratchathewi, 10400 Bangkok, Thailand; 2 Division of Rheumatology, Department of Pediatrics, Faculty of Medicine, Prince of Songkla University, Songkhla, Thailand; 3 Division of Allergy, Immunology, and Rheumatology, Department of Medicine, Faculty of Medicine Ramathibodi Hospital, Mahidol University, Bangkok, Thailand

**Keywords:** Transfer, Adolescence, Autoimmune, Pediatric, Independence, Skills

## Abstract

**Background:**

Most childhood-onset rheumatic diseases are chronic health conditions, which need long-term care throughout adulthood. A well-organized transition care is challenging and patient assessment of transition skills is needed for transfer preparation to an adult care setting. The Transition Readiness Assessment Questionnaire (TRAQ) is used to assess transition skills in chronically ill patients. Currently, limited transition skill assessment data exist in pediatric patients with rheumatic diseases, especially in Asian countries. This study aimed to determine the transition readiness skills in patients with rheumatic diseases and ascertain predictive factors contributing to high transition readiness skills.

**Methods:**

This is a cross-sectional study. All patients with rheumatic diseases aged 15–20 years were recruited. The TRAQ was cross-culturally adapted into the Thai language with good internal consistency and reliability. Patients completed the Thai TRAQ at the recent clinic visit and took the retest at a 2-week interval. Demographic data, baseline characteristics, clinical manifestations, and disease status were collected. Descriptive and logistic regression analyses were performed.

**Results:**

A total of 111 patients with a mean age of 17.4 ± 1.8 years were included. Median (IQR) disease duration was 6.4 (3.2–9.0) years. The most common rheumatic disease was juvenile idiopathic arthritis (48.6%), followed by systemic lupus erythematosus (35.1%). The mean TRAQ score was 3.85 ± 0.69. Independent visits (OR 4.35, 95% CI 1.23–15.37) was a predictor of a high TRAQ score. Furthermore, dependent visits (OR 7.84, 95% CI 2.41–25.50) was a predictor of low TRAQ score in the “appointment keeping” domain, whereas inactive disease (OR 4.54, 95% CI 1.25–16.55) was a predictor of a low TRAQ score in “tracking health issues” domain. Lack of knowledge and skills on health insurance coverage, financial management, appointment arrangement, and coping with their illness were issues causing lower TRAQ score.

**Conclusions:**

Patients, who had independent visits, had a higher chance to obtain higher TRAQ scores, whereas patients, who had an inactive disease or dependent visits, had less transition readiness skills. Physicians and parents should prepare to transfer patients to adult care settings, mainly encouraging independent living skills.

## Background

Most patients with childhood-onset rheumatic diseases have active disease and long-term morbidity throughout adulthood [1–7]. Failure to transfer patients into an adult care setting can lead to poor outcomes and increase morbidity and mortality; therefore, preparation for the healthcare transition process during the transfer period is vitally important [8–10]. “Transition of care” is defined as the purposeful and planned movement of adolescents and young adults with chronic physical and medical conditions from child-centered to adult-oriented healthcare systems [11]. Healthcare transition should maximize individual lifelong functioning by providing high-quality and developmentally appropriate healthcare services from adolescence to adulthood [12]. The recent data from systemic reviews had shown that a structured health care transition process could statistically improve the triple aim domains of population health (adherence to care monitoring, disease-specific measures, patient-reported health and quality of life, and self-care), patient experience of care (satisfaction), and utilization of care (utilization and process of care) outcomes in 16 (84%) from 19 studies [13]. The transition process should be initiated at the age of 12 years, and transfer should occur between the ages of 18 and 21 years by implementing the Six Core Elements of healthcare transition, including the assessment of transition readiness [14, 15].

There are several available transition readiness assessment tools, including the Transition Readiness Assessment Questionnaire (TRAQ) [16, 17], the TRxANSITION Scale [18], the STARx Questionnaire [19], the Am I ON TRAC for Adult Care questionnaire [20], TRANSITION-Q [21], and the Adolescent Assessment of Preparation for Transition (i.e., ADAPT) [22]. The TRAQ is an internally validated tool for a transition readiness assessment of self-management and self-advocacy, which are consistent with developmental skill acquisition during the transition period. The TRAQ guides healthcare providers on how to intervene and support skill acquisition [16, 23]. A systematic review of transition readiness assessment tools showed that the TRAQ has content validity, construct validity, and internal reliability [24]. A Cochrane Review in 2016 highlighted a few studies that used the TRAQ to assess transition readiness, but there was insufficient evidence certainty [25].

The American College of Rheumatology recommended that healthcare providers develop a transition of care protocol and support tools for patients with rheumatic diseases, including the TRAQ, to assess transition readiness [26]. Multicenter cohort studies of pediatric patients with the rheumatic disease during the transfer period showed that 60–80% of patients had active disease and 70–85% continued taking their medications. Furthermore, disease activity increased in 30% of patients, and 30% of patients missed their first adult rheumatology visit in the post-transfer period [9, 27]. Even though the TRAQ score did not significantly predict the time to transit to an adult care setting, a higher score showed a trend towards a shorter transition period compared with a lower score [28].

The TRAQ can help physicians to evaluate adolescents’ transition readiness before moving on to adult care systems. It can also enlighten healthcare professionals about unaccomplished skills that need to be facilitated before the transition. Because there is no standard transition assessment tool and no transition preparation process for patients with rheumatic diseases in Thailand, this study conducted a cross-cultural adaptation of the TRAQ into the Thai language. We aimed to determine the transition readiness skills in patients with childhood-onset rheumatic diseases and the predictive factors contributing to high transition readiness skills for better continuity of care in these patients.

## Methods

The study adopted a cross-sectional design. Patients aged 15–20 years followed up at the Pediatric Rheumatology clinic, Faculty of Medicine, Ramathibodi Hospital, Bangkok, Thailand, between 2008 and 2018 were included in the study. Patients were diagnosed with the following diseases: juvenile idiopathic arthritis (JIA), systemic lupus erythematosus (SLE), juvenile dermatomyositis (JDM), systemic sclerosis (SSc), overlap syndrome, mixed connective tissue disease, and primary systemic vasculitis, according to the International League Associations for Rheumatology classification of JIA [29], the Systemic Lupus International Collaborating Clinics classification criteria for SLE [30], Bohan and Peter diagnostic criteria for a definite diagnosis of JDM [31, 32], the Pediatric Rheumatology European Society/American College of Rheumatology/European League against Rheumatism provisional classification criteria for juvenile SSc [33], the classification and diagnostic criteria for mixed connective tissue disease [34], and the 2012 Revised International Chapel Hill Consensus Conference Nomenclature of Vasculitides [35], respectively. Overlap syndrome is defined as the presence of two or more distinctive features of rheumatic disease.

### TRAQ assessment tool

TRAQ version 5.0 is an internally validated transition readiness assessment tool with 20 self-administered questions in five domains regarding self-management and self-advocacy skills, including 1) managing medications, 2) appointment keeping, 3) tracking health issues, 4) talking with providers, and 5) managing daily activities. The question responses are scored on a scale of 1–5 as follows: 1) “no, I do not know how,” 2) “no, but I want to learn,” 3) “no, but I am learning to do this,” 4) “yes, I have started doing this,” and 5) “yes, I always do this when I need to.” These options were based on the stage of change of the transtheoretical model [23], including pre-contemplation, contemplation, preparation, action, and maintenance, which are consistent with the process of skills acquisition. Overall TRAQ scores are calculated using the average scores of 20 questions, and the TRAQ scores of each domain are calculated using the average scores of the questions in each domain. The overall TRAQ scores range from 1 to 5, which represent the stage of change.

### Development of the Thai TRAQ

At the beginning of the translation process, the author of the original questionnaire was contacted via email and granted permission for cross-cultural adaptation of the TRAQ to the Thai language. Translation was conducted according to international guidelines. Three independent translators performed the initial forward translation to the Thai language, and the forward translation was summarized in a meeting among expert pediatric rheumatologists and translators. Three independent translators performed backward translation to the English language. All versions of the translation were reviewed, and cross-cultural adaptation was performed according to the healthcare system’s context. The expert committee summarized the pre-final version of the translated questionnaire with semantic and conceptual equivalence. Comprehensibility of the pre-final version was tested in a pilot sample of 10 patients with chronic rheumatic diseases. Questions with less than 80% agreement of comprehensibility were revised, and the final version was reviewed and approved by the expert committee.

### Data collection

All patients completed the Thai TRAQ at the recent clinic visit and the retest was performed at a 2-week interval [36, 37]. Demographic data and clinical characteristics, including diagnosis, disease duration, disease activity, medications, education level, health insurance, family status, and socioeconomic status, were collected from medical records. Inactive disease status was defined according to disease-specific criteria, including Wallace’s criteria for JIA [38], the SLE disease activity index [39, 40], Pediatric Rheumatology International Trials Organization criteria for JDM [41], and the Pediatric Vasculitis Activity Score (PVAS) for primary systemic vasculitis [42, 43].

### Statistical analysis

Performance of the Thai TRAQ was validated by assessment of internal consistency using Cronbach’s alpha coefficient and test-retest reliability using the intraclass correlation coefficient (ICC). Cronbach’s alpha values < 0.5 were considered unacceptable, values between 0.5 and 0.59 were considered poor, values between 0.6 and 0.69 were considered questionable, values between 0.7 and 0.79 were considered acceptable, values between 0.8 and 0.89 were considered good, and values between 0.9 and 1.0 were considered excellent. ICC values < 0.4 indicated poor reliability, values between 0.4 and 0.59 indicated fair reliability, values between 0.6 and 0.74 indicated good reliability, and values between 0.75 and 1.0 indicated excellent reliability. Categorical data were presented as frequencies and percentages. Continuous data were presented as means and standard deviations or medians and interquartile ranges, as appropriate. The Student’s t-test or one-way analysis of variance were used for comparisons between groups. Logistic regression analysis was performed to analyze predictors of high and low TRAQ scores. Statistical significance was defined as a *P*-value of < 0.05. Stata version 15 (StataCorp, College Station, TX, USA) was used for statistical analysis.

## Results

### Demographic data and clinical characteristics

A total of 111 patients from the pediatric rheumatology clinic participated in the study. The majority of patients were female and the most common diagnosis was JIA, followed by SLE, and vasculitis (Table 1). Most patients in this study remained in an active disease state (77.5%) and continued taking at least one medication (91.9%), including steroid (34.2%), disease-modifying anti-rheumatic drugs (DMARDs) (80.2%), or biologic therapy (10.8%). Around 80% of patients were studying in high school, whereas the remainder studied for a bachelor’s degree. Regarding health insurance, half of the patients were enrolled on the Universal Coverage Scheme (UCS), 21.6% were enrolled on the Civil Servant Medical Benefit Scheme (CSMBS), around 20% had no health insurance, and the remaining patients had private or company health insurance or were receiving disability benefits. Only some patients independently attended the clinic without a caretaker (18.9%). Around 16.2% of patients had a prior discussion about transition processes and policies, and 25.2% were worried about the transition to an adult setting.

**Table 1:** Demographic data of children with rheumatic diseases

Variables	Number (%)
Age (years)^a^	17.4 ± 1.8
Female	79 (71.2)
Disease duration (years)^b^	6.4 (3.2–9.0)
Level of education	
High school	90 (81.1)
Bachelor’s degree	21 (18.9)
Type of health insurance	
Universal Coverage Scheme	57 (51.4)
Civil Servant Medical Benefit Scheme	24 (21.6)
Company health insurance	3 (2.7)
Private health insurance	3 (2.7)
Disability benefit	1 (0.9)
None	22 (19.8)
Family status	
Living with both parents	88 (79.3)
Living with other relatives in the same household	41 (36.9)
Diagnosis	
Juvenile idiopathic arthritis	54 (48.6)
Systemic lupus erythematosus	39 (35.1)
Scleroderma	1 (0.9)
Juvenile dermatomyositis/inflammatory myositis	4 (3.6)
Overlap syndrome	5 (4.5)
Mixed connective tissue disease	2 (1.8)
Systemic vasculitis	6 (5.4)

^a^Data are presented as mean ± standard deviation, ^b^ median (interquartile range)

### Performance of the Thai TRAQ

Two questions in the “managing medications” and “appointment keeping” domains were adapted according to real practice and the Thai healthcare system. Question number 1 (“Do you fill the prescription if you need to?”) was adapted to “Can you take a drug prescription to get the medications if you need to?” Question number 9 (“Do you apply for health insurance if you lose your current coverage?”) was adapted to “Can you get the Universal Coverage Scheme or find other health care programs if your current health care program ends?” The pre-final version’s comprehensibility in the pilot sample was at least 80% agreement for all questions, and the final version was reviewed and approved by the expert committee with 100% agreement. All patients completed the TRAQ at the enrollment visit, and 105 patients completed the TRAQ retest at a 2-week interval. Two rheumatologists assessed content validity with 100% agreement, whereas criteria validity could not be tested because of the unavailable standard readiness assessment tool. The Thai TRAQ showed excellent internal consistency (Cronbach’s alpha = 0.90) and a good-to-excellent reliability (ICC = 0.60–0.89; Table 2). The translation and cross-cultural adaptation of TRAQ from the original version to the Thai version is shown in Table 3.

**Table 2:** Overall TRAQ scores and TRAQ scores in each domain

Question	TRAQ scores (*n* = 111)	Intraclass correlation coefficient (*n* = 105)
Managing medications	4.26 ± 0.73	
Question 1	4.41 ± 0.92	0.75 (0.63–0.83)
Question 2	3.98 ± 1.18	0.83 (0.74–0.88)
Question 3	4.76 ± 0.65	0.60 (0.41–0.73)
Question 4	3.90 ± 1.13	0.73 (0.60–0.82)
Appointment keeping	3.42 ± 0.99	
Question 5	3.42 ± 1.34	0.80 (0.70–0.86)
Question 6	4.00 ± 1.27	0.75 (0.63–0.83)
Question 7	4.11 ± 1.17	0.81 (0.72–0.87)
Question 8	3.48 ± 1.41	0.85 (0.78–0.90)
Question 9	3.12 ± 1.43	0.73 (0.60–0.81)
Question 10	2.81 ± 1.25	0.67 (0.52–0.78)
Question 11	3.04 ± 1.47	0.80 (0.71–0.87)
Tracking health issues	3.47 ± 0.78	
Question 12	4.19 ± 1.12	0.73 (0.61–0.82)
Question 13	3.76 ± 1.22	0.77 (0.66–0.84)
Question 14	3.43 ± 1.24	0.76 (0.65–0.84)
Question 15	2.50 ± 1.48	0.83 (0.75–0.88)
Talking with providers	4.62 ± 0.63	
Question 16	4.57 ± 0.76	0.65 (0.48–0.76)
Question 17	4.68 ± 0.69	0.77 (0.66–0.84)
Managing daily activities	4.31 ± 0.76	
Question 18	3.92 ± 1.16	0.83 (0.75–0.89)
Question 19	4.39 ± 0.87	0.89 (0.84–0.93)
Question 20	4.63 ± 0.73	0.70 (0.56–0.80)

**Table 3:** Translation and cross-cultural adaptation of Transition Readiness Assessment Questionnaire (TRAQ) from the original version to the Thai version

Original version	Thai version
***Managing Medications***	***Managing the medicine***
1. Do you fill a prescription if you need to?	1. Can you take a drug prescription to get the medications if you need to?
2. Do you know what to do if you are having a bad reaction to your medications?	2. Do you know what you should do if there are abnormal symptoms from using the medicine?
3. Do you take medications correctly and on your own?	3. Can you take medicine correctly by yourself?
4. Do you reorder medications before they run out?	4. When the medicine runs out, can you ask for more medicine from the doctor by yourself?
***Appointment Keeping***	***Managing the appointment with the doctor***
5. Do you call the doctor’s office to make an appointment?	5. Can you call the hospital to make an appointment with the doctor?
6. Do you follow-up on any referrals for tests, check-ups or labs?	6. Can you go for a check-up or a blood test at a hospital near your house if the doctor asks you to do it?
7. Do you arrange for your ride to medical appointments?	7. Can you manage the travel to meet the doctor as appointed?
8. Do you call the doctor about unusual changes in your health (For example: Allergic reactions)?	8. Do you make a phone call to consult with the doctor when there are abnormal symptoms with your health, for example, drug allergies?
9. Do you apply for health insurance if you lose your current coverage?	9. Can you get the Universal Coverage Scheme or find other health care programs if your current health care program ends?
10. Do you know what your health insurance covers?	10. Do you know what your health care rights cover?
11. Do you manage your money & budget household expenses (For example: use checking/debit card)?	11. Do you manage your finance and expenditure by yourself (for example, using a debit or credit card)
***Tracking Health Issues***	***Health follow-up***
12. Do you fill out the medical history form, including a list of your allergies?	12. Have you written down information regarding your health, including information regarding what you are allergic to?
13. Do you keep a calendar or list of medical and other appointments?	13. Have you written down in your calendar or note the information regarding the use of the medicine and appointments to see the doctor?
14. Do you make a list of questions before the doctor’s visit?	14. Do you prepare questions before going to see the doctor?
15. Do you get financial help with school or work?	15. Do you receive financial support for education or work?
***Talking with Providers***	***Conversation with medical staff***
16. Do you tell the doctor or nurse what you are feeling?	16. Have you told the doctor or nurse how you feel or what symptoms you have?
17. Do you answer questions that are asked by the doctor, nurse, or clinic staff?	17. Can you answer questions asked by the doctor, the nurse, or hospital staff?
***Managing Daily Activities***	***Managing daily activities***
18. Do you help plan or prepare meals/food?	18. Do you help in planning for cooking or meal preparation?
19. Do you keep home/room clean or clean-up after meals?	19. Do you clean the house/room or clean up the table after meals?
20. Do you use neighborhood stores and services (For example: Grocery stores and pharmacy stores)?	20. Can you go to buy something, or use the service of shops nearby to your house by yourself?

### TRAQ scores in each domain

The mean TRAQ score in this study was 3.85 ± 0.69. Questions related to health insurance coverage (question 9, 10), financial management (question 11), and financial support (question 15) had the lowest TRAQ scores out of all the questions, followed by questions related to appointment management (question 5), doctor consultation when unusual changes occur (question 8), and questions with doctors about health issues (question 14) as shown in Table 2. Because all questions above had low TRAQ scores, resulting in the low average of TRAQ scores (< 4.0) of appointment keeping (question 5–11) and tracking health issues (question 12–15) domains. Comparisons of mean TRAQ scores in various parameters, including age, sex, educational level, family status, disease status, medications, and transition variables, are shown in Table 4. Patients had significantly higher TRAQ scores if they aged > 18 years and were studying for a bachelor’s degree. Patients who had previously discussed the transition with the physicians and could independently visit the clinic by themselves also had significantly higher TRAQ scores.

**Table 4:** Comparison of TRAQ scores in various parameters (*n* = 111)

Parameters	TRAQ score (mean ± SD)	*P*-value
Age		< 0.001*
< 18 years (*n* = 78)	3.70 ± 0.72	
≥ 18 years (*n* = 33)	4.22 ± 0.40	
Sex		0.51
Male (*n* = 32)	3.92 ± 0.82	
Female (*n* = 79)	3.83 ± 0.63	
Education level		< 0.001*
Below bachelor’s degree (*n* = 90)	3.76 ± 0.71	
Attending bachelor’s degree (*n* = 21)	4.25 ± 0.34	
Family status		0.44
Living with both parents (*n* = 88)	3.83 ± 0.65	
Living with one parent (*n* = 17)	3.87 ± 0.91	
Not living with parents (*n* = 6)	4.20 ± 0.50	
Disease status		0.69
Active (*n* = 86)	3.87 ± 0.71	
Inactive (*n* = 25)	3.81 ± 0.62	
Medication		0.33
At least one medication (*n* = 102)	3.87 ± 0.68	
Free of medication (*n* = 9)	3.64 ± 0.76	
Diagnosis		0.30
Connective tissue disease (*n* = 50)	3.85 ± 0.55	
Juvenile idiopathic arthritis (*n* = 54)	3.82 ± 0.82	
Primary vasculitis (*n* = 7)	4.17 ± 0.36	
Transition discussion		0.045*
Yes (*n* = 18)	4.15 ± 0.56	
No (*n* = 93)	3.80 ± 0.69	
Transition anxiety		0.90
Yes (*n* = 28)	3.84 ± 0.81	
No (*n* = 83)	3.86 ± 0.64	
Independent visits		0.001*
Yes (*n* = 21)	4.28 ± 0.56	
No (*n* = 90)	3.76 ± 0.68	

*P < 0.05 indicates statistical significance

### Predictors of high and low TRAQ scores

A mean TRAQ score of ≥4.0 was compatible with the action stage in the stage of change model, which means that patients had already started acquiring transition skills. Patients were classified into two groups: 1) patients with high TRAQ scores of ≥4.0 or 2) patients with low TRAQ scores of < 4.0. The univariate analysis showed four significant predictors for high TRAQ score, including 1) age > 18 years, 2) studying for a bachelor’s degree, 3) transition discussion, and 4) independent visits. However, multivariate analysis demonstrated a significant predictor only “independent visits” with an odds ratio [OD] of 4.35 (95% confidence interval [CI] 1.23–15.37), as shown in Table 5. Furthermore, this study analyzed predictors of low TRAQ scores in two domains: 1) appointment keeping and 2) tracking health issues because both domains had mean TRAQ scores of < 4.0. Predictors of low TRAQ score in the “appointment keeping” domain was dependent visits (OR 7.84, 95% CI 2.41–25.50), whereas inactive disease (OR 4.54, 95% CI 1.25–16.55) was a predictor of low TRAQ score in “tracking health issues” domain as shown in Table 6.

**Table 5:** Predictors of a high TRAQ score (cut off > 4)

Factors	Univariate analysis			Multivariate analysis		
	Odds ratio	95% CI	*P*-value	Odds ratio	95% CI	*P*-value
Age ≥ 18 years	4.04	1.66–9.85	0.002*	1.34	0.40–4.49	0.639
Male	1.46	0.64–3.34	0.370			
Studying for a bachelor’s degree	5.82	1.81–18.68	0.003*	2.88	0.64–13.04	0.170
Disease duration	1.08	0.97–1.21	0.142			
Active disease status	1.08	0.44–2.64	0.860			
Transition discussion	4.44	1.36–14.51	0.014*	2.32	0.60–8.98	0.224
Transition anxiety	1.83	0.77–4.38	0.175			
Independent visits	5.82	1.81–18.68	0.003*	4.35	1.23–15.37	0.022*

*P < 0.05 indicates statistical significance

**Table 6:** Predictors of a low TRAQ score (cut off < 4)

Factors	Univariate analysis			Multivariate analysis		
	Odds ratio	95% CI	*P*-value	Odds ratio	95% CI	*P*-value
**Low TRAQ score in domain 2 (Appointment Keeping)**						
Age < 18 years	3.48	1.48–8.17	0.004*	1.40	0.40–4.92	0.595
Studying below a bachelor’s degree	3.28	1.24–8.72	0.017*	1.88	0.47–7.57	0.376
Dependent visits	9.89	3.25–30.11	< 0.001*	7.84	2.41–25.50	0.001*
**Low TRAQ score in domain 3 (Tracking Health Issues)**						
Inactive disease status	4.80	1.33–17.27	0.017*	4.54	1.25–16.55	0.022*
Dependent visits	2.71	1.03–7.14	0.044*	2.51	0.93–6.82	0.071

*P < 0.05 indicates statistical significance

## Discussion

This study developed a valid and reliable transition readiness assessment tool for Thai adolescents with rheumatic diseases and identified predictors for high and low TRAQ scores. The results demonstrated that these patients’ weaknesses were the skills in the appointment keeping and tracking health issues domains. These two domains, which had low TRAQ scores, comprise knowledge of health insurance, the skills in financial management, financial support, appointment management, and coping and concerning about their health-related issues. Patients who were > 18 years of age, studying for a bachelor’s degree, having transition discussions with their physicians, and attending independent visits, had significantly higher TRAQ scores. However, a predictor of high transition readiness skills was only attending independent visits. Furthermore, dependent visits was a predictor of low TRAQ score in the appointment keeping domain, whereas inactive disease was a predictor of low TRAQ score in tracking health issues.

Some questions were adapted to ensure compatibility with our healthcare system (e.g., question number 1, which is related to the prescription filling process). The process of receiving medications in Thailand is different from Western countries. In the Thai healthcare system, patients receive medications at the hospital after leaving the doctor’s office. Hospital pharmacies are the only places permitted to dispense specific drugs, especially immunosuppressive medications and biologic agents. Patients cannot get these medications from outside pharmacies. Therefore, question number 1 was adapted to assess the process of obtaining medications from pharmacists at the hospital. It might be more comfortable for patients to obtain medicines in Thailand than in Western countries, but this is the first step toward patient independence and taking responsibility for their health.

Another question that was adapted was question number 9, which is related to health insurance. The majority of Thai citizens can apply for the UCS from the government at no cost, so they usually do not apply for other health insurance programs. The UCS covers most basic and immunosuppressive drugs used to treat rheumatic diseases, except biologic agents. However, the UCS has a short expiration date, and most patients need to reapply before attending their subsequent clinic visits. Patients whose parents work for the government or state enterprise are eligible for the CSMBS until they are 20 years old. This benefit covers all immunosuppressive medications and some biologic agents. After 20 years of age, these patients need to apply for other healthcare benefits, including the UCS or other health insurance programs. Therefore, question number 9 was adapted to “Can you get the UCS or find other health care programs if your current health care program ends?” TRAQ can be used in different cultures and lifestyles. Only some questions should be modified according to the context of the health care system and financial management in each region.

The results of this study show that the percentage of patients with an active disease status (78%) was similar to previous studies (60–80%), but the percentage of patients that took at least one medication (93%) was higher compared with previous studies (70–85%) [9, 27]. Because most patients in our study had JIA or SLE, they needed long-term treatment until adulthood. Thailand has only a small number of pediatric rheumatologists; thus, a delay in patient referral to pediatric rheumatologists is expected, resulting in poor outcomes and the need for long-term medications. Additionally, our patients could not access biologic therapy as soon as indicated, which is different from developed countries’ situation. Thus, these patients still need DMARDs for long-term disease control.

Regarding the association between TRAQ score and specific diseases, previous studies have demonstrated that patients with mental illness or developmental disabilities tended to have lower TRAQ scores than patients with chronic diseases of physical function [16]. In this study, our patients were affected by physical disability, not by mental illness; therefore, the TRAQ scores in each rheumatic disease were not statistically different. The hypothesis that TRAQ scores were higher in patients with rheumatic diseases without co-existing mental illness compared with patients with co-existing mental illness is supported by the TRAQ scores observed in our study and Anelli et al. study [44]. Both studies demonstrated higher TRAQ scores than other studies [16, 45], which assessed patients with cognitive impairment and mental health issues. Moreover, a previous study showed that TRAQ scores in patients without intellectual and development disabilities were not statistically different, although they had different types of diseases, including rheumatic, endocrine, and gastrointestinal conditions [28].

In terms of diseases that affect adolescent development, such as epilepsy, patients with epilepsy tend to have barriers to knowledge-related disease, psychosocial and cognitive function [46]. However, a recent study focusing on the factors related to transition readiness skills in epilepsy patients showed that age was the only predictor of transition readiness [47]. For a chronic disease like diabetes mellitus, a Korean study demonstrated that factors associated with transition readiness were self-management competency and age [48]. Nevertheless, a study from the United States suggested that provider communication, parent knowledge/monitoring, and friend knowledge/helpfulness were associated with higher transition readiness skills [49]. These findings might reflect that factors associated with transition readiness skills are varied depending on the type of disease, culture, and parenting style. Therefore, we should interpret the studies with caution, and patients’ characteristics should be considered when applying the data to our patients.

The knowledge and skills related to health insurance coverage and financial management had the lowest TRAQ scores. Because most of the patients in this study were of low socioeconomic status, most of them applied for the UCS. Patients’ parents/caregivers reapplied on their behalf, although these patients were young adults. Differences exist between Thai culture and Western culture in terms of living status and financial management. Most Thai children live with their parents, even as young adults, and they only leave their parents once they are married. Furthermore, most of them do not have a part-time job and do not undertake extra work while attending university. Therefore, health insurance and healthcare financing are managed by parents or caregivers.

The other concern from the results of this study is the skills related to coping and concerning patients’ health issues (based on Question 8, 14). Achieving these skills, patients should have the responsibility for their healthcare and decision-making [50]. Since adolescents have to develop self-concept and identity, the development of the illness during this period may be complex for them to integrate the disease into their evolving sense of self. It might appear to the physicians that these adolescents do not have an awareness and concern of the disease. Many teenagers with chronic illness may feel that they cannot control their life because of the illness [51]. Physicians should understand this situation and allow these patients to participate in the treatment decision. Patients in late adolescence or young adults also need support from their parents and physicians. Preparing the patients for these skills should begin early and before transfer to adult care settings [50]. Because only 16% of patients in this study had a prior discussion about transition processes and policies, discussing these issues as part of transition sessions is essential.

Regarding good transition readiness, independent visits was only a predictor of high TRAQ scores in this study. Adolescence is time to develop physical, pubertal, cognitive maturation, including the development of independent living skills. Therefore, we expected adolescents to take responsibility for their health care, including independent visits. However, stressful situations such as an illness may cause them to regress and fail to cope with their health issues [51]. Another factor that affects independence is parenting style. Parents in Thai culture always overprotective of their children, even as young adults. This parenting style can cause adolescents to delay the ability to take responsibility for their illness. A current review showed that lack of patient independence in Asian culture was a concern for transferring patients to adult care settings [52]. Parents and families in Asian culture believed that taking care of children with chronic diseases was the family responsibility, which is different from the Western culture [53]. Therefore, teaching these patients to be more independent, the parents and families should change their perspective and support the transition process.

Although older age and high education level were not a predictor of high TRAQ score, older patients or patients studying for a bachelor’s degree tended to have higher TRAQ scores than younger ones or patients studying below bachelor’s degree. According to normal adolescent development, older adolescents should have more cognitive maturation, especially independent living skills, than younger ones. Studying for a bachelor’s degree should help adolescents learn how to become independent and have more responsibility for themselves. Universities may offer varieties of experiences that enhance adolescents’ maturity and increase cognitive function [54]. A previous study showed that educational level was associated with five of the six quality of life domains, including independence [55]. Due to a small sample size of patients aged > 18 years and patients studying for a bachelor’s degree in this study, the statistical analysis of the predictors of high TRAQ scores might be underestimated. Further study with the larger population on this age group is recommended.

Focusing on the lower mean scores in the domains of appointment keeping and tracking health issues, we found that dependent visits was a predictor of low TRAQ scores in the appointment keeping domain, whereas inactive disease was a predictor of low TRAQ scores in the tracking health issues domain. The previous study demonstrated that independent visits correlated with successful transfer to adult care settings [56]. Since independent visits reflects the independent living skills and self-management, it is predictable that dependent visits could be one factor associated with low skills on appointment arrangement, financial management, and coping with their illness. Regarding the predictor of low TRAQ scores on tracking health issues, patients with inactive disease tended to be less concerned about their health issues from our clinical practice interview. The previous study suggested that physicians should re-evaluate the patients’ disease-related knowledge if the disease occurred when they were young [51].

From our study’s results, we recommend physicians and parents to prepare the transition readiness skills before transferring patients to adult care settings, especially providing knowledge on patient conditions and independent living skills. The previous literature advised physicians to create a private conversation with adolescent patients and allow them to participate in their transition process [50, 57, 58]. In this way, patients can discuss their conditions and concerns with their physicians, leading to increased independence and control. Moreover, physicians should routinely ask their patients about their preference for independent visits [58]. Parents and families should also promote independence and autonomy for their children. A multidisciplinary team, including physicians, nurses, and school teachers, should encourage parents and families to gradually hand over responsibility to their children in self-management, including taking the medication, managing appointments, making a treatment decision, and having independent visits. Physicians should also clarify the disease course and future treatment plan with the parents before the transition because parental concerns about their children’s conditions can lead to a negative perception of the disease and overprotection of children [59]. Consistent with findings from previous literature [16, 60], lack of understanding about health insurance was also an essential issue. Therefore, anticipatory guidance about health insurance before transfer to adult care settings is recommended.

To the best of our knowledge, this is the first study carried out in Thailand using the cross-cultural adapted TRAQ to assess transition readiness in adolescents with rheumatic diseases. However, this study had some limitations. First, this study was performed at a single center; thus, it might not represent the whole Thai adolescent population. Nevertheless, our hospital is the main referral center in Thailand, which receives patients from all over the country. Second, several factors associated with transition readiness, including psychological factors, strong relationships with pediatricians, and parenting style, were not assessed. Therefore, further multicenter studies with prospective cohort designs, including psychological factor assessments, are recommended.

In summary, the Thai TRAQ is a valid and reliable tool with good performance to assess transition readiness in Thai adolescents with rheumatic diseases. Patients, who independently attended clinic visits, have a high transition readiness skill. Practitioners should facilitate patient transfer from pediatric to adult care settings by preparing patients for this transition, mainly focused on the knowledge on health conditions, independent living skills, and anticipatory guidance regarding health insurance. Moreover, it is essential to raise awareness of the disease to the patients and re-evaluate their disease-related knowledge, especially during inactive disease. Parents should have the leading role in teaching and encouraging their children about independent living skills. Well-planned transitional care may help adolescents to achieve a successful transition and achieve good health and wellbeing.

## Data Availability

The datasets analyzed during the current study are not publicly available due to confidentiality agreement of the data with the participants but are available from the corresponding author on reasonable request.
